# Expression of Concern: Time-to-recovery after cesarean section delivery among women who gave birth through cesarean section at Hawassa University Comprehensive Specialized Hospital, South Ethiopia: A prospective cohort study

**DOI:** 10.1371/journal.pgph.0004983

**Published:** 2025-07-28

**Authors:** The PLOS Global Public Health Editors

The *PLOS Global Public Health* Editors issue this Expression of Concern to alert readers that this article [1] was identified as one of a series of submissions for which we have concerns about authorship and adherence to the journal’s first and second publication criteria. We regret that these issues were not addressed prior to the article’s publication.

Contrary to the declaration in the Data Availability statement, the original raw data files supporting the article’s results were not provided with the article, and the authors have not provided these data following editorial request. As the original raw data files supporting the article’s results have not been made available, this article [1] does not comply with the PLOS Data Availability policy.
