# Supplementary material for: Time-to-recovery after cesarean section delivery among women who gave birth through cesarean section at Hawassa University Comprehensive Specialized Hospital, South Ethiopia: A prospective cohort study
Source: PLOS Glob Public Health. 2022 Oct 5;2(10):e0000696. doi: 10.1371/journal.pgph.0000696 (PMC10022236; doi:10.1371/journal.pgph.0000696)
Supplement: Attachment — Submitted filename: 1. Responses to the reviewers.docx [file pgph.0000696.s001.docx]

***17 March, 2022***

**Dear Dr**. Charles Anawo Ameh

Academic Editor

PLOS Global Public Health

***Ref: A point-by-point response to reviewers comments on Manuscript reference number* [PGPH-D-21-00438]**

***Reviewers’ comments***

**Dear reviewer 1**, First of all I would like to thank you very much for your motivating, inspiring and astonishing appreciation towards our manuscript. We are also indebted for your constructive and insightful comments which we believe that it would improve the quality of our manuscript. Thus, according to your comments, and suggestions the following amendments has been taken.

**Q1. Although indeed this is a prospective study with a cohort of women, this is not a cohort study because there is no clear exposure defined.**

**Response:** Thank you very much for your comment and concern regarding the use of the study design. In our study we used an internal comparisons group. So, we have categorized study participants in terms of their specific exposure status (ANC visit Yes/No, Hypertension Yes/No, Diabetes mellitus Yes/No, Previous history of CS Yes/No, Types skin incision Transverse/Vertical …etc….) and then compared the time-to-recovery ***after cesarean section delivery*** in exposed individuals with the time-to-recovery after cesarean section delivery in on-exposed in individuals.

Q2. Why the Kolmogorov Smirnov test for normality. We do not need this.

**Response: Thank you for your comment. We removed it according to your comment.**

Q3. On the results and discussion, it would be better to remove the “±” on the SD.

**Response:** We have removed “±” on the SD.

Q4. Table 5 - the “abortion” variable sounds to be “previous abortion” correct?

**Response: Thank you very much for your insights. We have corrected as** “previous abortion”.

Q6. Why age was dichotomized? And please use as reference the category with more observations

**Response:** The age was dichotomized for the ease of the comparison in the regression. Thank you we replaced the reference category with more observations.

**Q7. Table 6:** How the means were computed? Does the method used here account for censoring? It seems that these are unadjusted mean recovery times. I imagine computing the adjusted ones but state that these are unadjusted.

Response: We have corrected based on your concern.

**Q8. There are English issues.**

**Response: Thank you once again for such wonderful comments. We have corrected it all.**

**Dear reviewer 2**, First of all I would like to thank extends my deep indebtedness for your beneficial, insightful and positive comments which i believe that it would improve the quality of the manuscript.

Q1**. Gross grammatical errors noted. This would have been resolved with a grammar checking app before submission. Revisions attached.**

**Answer:** I did through and in-depth editing in line with your comments.
