# Supplementary material for: Time-to-recovery after cesarean section delivery among women who gave birth through cesarean section at Hawassa University Comprehensive Specialized Hospital, South Ethiopia: A prospective cohort study
Source: PLOS Glob Public Health. 2022 Oct 5;2(10):e0000696. doi: 10.1371/journal.pgph.0000696 (PMC10022236; doi:10.1371/journal.pgph.0000696)
Supplement: Attachment — Submitted filename: 3. Responses to the reviewers.docx [file pgph.0000696.s002.docx]

***22 June, 2022***

**Dear** **Lucinda Shen, MSc**

**Staff Editor**

**PLOS Global Public Health**

***Ref: A point-by-point response to reviewers comments on Manuscript reference number* [**PGPH-D-21-00438R1

***Reviewers’ comments***

**Dear reviewer 1**: I am indebted for your constructive, heedful, and insightful comments which we believe that it would improve the quality of our manuscript. Thus, according to your comments, and suggestions the following amendments has been taken.

**Q1. In the response letter, the authors state that they removed the Kolmogorov Smirnov test for normality. They did not. It is very clear in the 3^rd^ line of the “data processing and analysis”.**

- **Response:** Thank you very much. I have removed it.

**Q2. Please make sure it is written Epi Info (not EPINFO), and please add a citation. Did you use indeed two different versions (in the population size version 7; and the analysis some another version).**

Response: ***Thank you for your comment. It was an editorial error; we did use Epi Info the same version. I have cited***

**Q3. I believe the confusion starts from the unclear labeling of the columns in table 4. The last column “cumulative proportion surviving at end of interval”, although in the survival analysis language is indeed survival, to avoid confusion I suggest to call it “cumulative proportion of not recovered”.**

Response: Really!!!!! I would like to extend my deepest appreciation and respect for your critical and heedful comment. So I have definitely accepted and made it “cumulative proportion of not recovered”.

**Q4. The first 2 citations are reports. Right? Please make sure they are correctly written.**

Response: Thank you. I have corrected accordingly

**Q5. In the background the first line of the 3^rd^ paragraph please clarify why the increasing rate of CS delivery becomes a major public health concern.**

**Response:** Thanks. I have briefly described the reason why CS delivery becomes a major public health problem.
